# Our New York Letter

**Published:** 1892-06

**Authors:** 


### Correspondence.

### NEW YORK LETTER.

NEW YORK, April 19, 1892.

Two papers were read last night by Drs. L. Duncan Bulkley and C. W. Allen before the Section of Public Hygiene at the New York Academy of Medicine on the frequency, contagiousness and prophylaxis of syphilis. Both papers were precisely of the same character and in them high colored and exaggerated views of the destructiveness of this disease were put forth by the authors. Statistics were read showing the great percentage with which syphilis prevailed in some countries of Europe, emphasizing its extreme prevalence in certain parts of Russia, Portugal and France, and calling attention to the legislative action its great frequency and contagiousness called forth. The authors suggested that similar legislation be applied to restrict the disease in this country where it was becoming more and more prevalent every year.

A number of well known syphilographers were present at the meeting and from the character of the discussion which followed, it was clear that the picture drawn by the authors of the two papers did not harmonize with their views of the destructiveness or frequency of this disease.

Dr. E. L. Keyes, who opened the discussion, ridiculed some of the views expressed therein and stated that in his opinion gonorrhœa and its complications killed more people than syphilis. The vast majority of people who acquire syphilis late in life do not seriously suffer from it, if in reasonable good health before acquiring it, and the disease does not materially affect their life, health and happiness. The bad cases are those who neglect its treatment, who drink excessively and have certain idiosyncrasies. The bad symptoms of the acquired form of syphilis are not due to the disease, but to the soil. He has seen several members of the same family who had acquired syphilis at different periods of life who developed a most malignant and vicious form of the disease, while in the case of others it amounted to practically nothing. It was not in either case a good or a bad syphilis, but the soil in one instance was capable of producing luxurious fruit, and in the other none at all. There was no doubt in his mind that gonorrhœa ultimately kills more people than syphilis. It is more contagious, more insiduous, harder to detect, capable of stirring up a fresh gonorrhœal capacity and reproducing the contagion, ending finally in stricture, kidney disease and death. It destroys the population by producing salpyngitis and preventing conception. If legislation were to cover venereal disease at all, he thought they should carry it out so as to include gonorrhœa.

Dr. Sturgis also agreed with Dr. Keys as to the greater danger of gonorrhœa in its sequelæ than syphilis. With regard to legislation, there is nothing he knew which would stop syphilis, and the moment they attempted legislation to that end they would start such a hornets nest about their heads that they wished they had not made the attempt. He, the speaker, has told men who had disease, not to kiss their wives, and a few weeks later the wives came to his office with the same trouble. So it goes, and so it will continue to go till the end of time, and we cannot prevent it. All that can be done is to advise, but so far as the question of legislation was concerned, he believed it to be wholly powerless.

Last week a young woman with an exfoliative dermatitis presented herself at the skin class of the North Western Dispensary. The disease had lasted for a period of three months and the entire surface of the body from hair to toe nails was affected. The skin was reddened, the natural marking exaggerated and the whole body covered with large, semi-adherent epithelial scales, especially marked on the flexor aspect of the limbs. The patient was extremely neurotic, had marked hysterical attacks, and was peculiarly susceptible to arsenic, which is a specific in this affection. The arsenic was so badly borne in this case—an interesting feature of the malady—that five drop doses of Fowler's solution produced symptoms of poisoning. The treatment, therefore, had to be confined exclusively to external emollient applications.

A feature of the skin class of this same dispensary, is the the large number of colored women who come with late secondary lesions of syphilis. Syphilis in the negro, like other skin affections, is hard to diagnosticate. The difficulty of diagnosis is due to the masking of the color by the dark hue of the patient's skin. The diagnosis is to be made on the shape and location of the lesion. It is generally in the form of a papular eruption confined to the face and head. It begins as a dark papule, spreads peripherically and leaves a deeply pigmented and slightly atrophied centre. The circles thus formed coalesce into large, gyrating figures.

In a paper on "Hydrotherapy," read before the New York Academy of Medicine, Dr. Wm. H. Draper sums up with the following conclusions:

(1.) In bath cures, the beneficial effects are due to the thermal qualities of the water, there being no evidence that absorption plays any part in it.

(2.) The chloride of sodium springs are chiefly useful for bathing and drinking purposes in anæmia and chronic rheumatism, and in disordered digestion; also, in nervous and chronic catarrhal affections.

(3.) That the alkaline and soda springs are mainly beneficial in gouty affections.

(4.) That the sulphur springs may be regarded as indifferent waters.

(5.) That the iron springs may prove useful in simple anæmia.

Dr. S. Baruch, in discussing the paper, said that the external use of water he considered to be one of the most potent remedies that we have to-day. A remedy to be useful must possess three qualities:

1. Its action must be explicable upon a rational basis; 2. It must be adaptable to exact dosage; 3. It must stand the test of clinical experience.

All these qualities are possessed by hydrotherapy. Its influence upon the nervous system by an excitation of the peripheral nerves, followed by a contraction or dilatation of the blood vessels, has been shown by experiments made by Dr. Max Schuller, who trephined a rabbit and exposed the pia mater. On putting a cold compress on the rabbit, the vessels of the pia mater were seen to dilate, while, on the other hand, when a warm compress was used, the vessels contracted.

He, the speaker, has cured a number of desperate cases of insomnia by carrying out the idea shown in this experiment.

At the Montifore Home, in this city, they have succeeded in restoring to their families individuals who have been sent there from other hospitals to die, and who, by means of hydrotherapy have been restored to perfect health.

This treatment had not become popular owing to the prejudice of the people, and when the physician is wholly convinced of the value of this method, and understands how to apply it, this prejudice will be overcome.

